# Human Computation as a New Method for Evidence-Based Knowledge Transfer in Web-Based Guideline Development Groups: Proof of Concept Randomized Controlled Trial

**DOI:** 10.2196/jmir.2055

**Published:** 2013-01-17

**Authors:** Annemie Heselmans, Bert Aertgeerts, Peter Donceel, Stijn Van de Velde, Peter Vanbrabant, Dirk Ramaekers

**Affiliations:** ^1^School of Public Health and Primary CareKatholieke Universiteit LeuvenLeuvenBelgium; ^2^School of Public Health and Primary CareAcademic Center for General PracticeKatholieke Universiteit LeuvenLeuvenBelgium; ^3^Belgian Center for Evidence-Based MedicineBelgian Branch of the Cochrane CollaborationLeuvenBelgium; ^4^School of Public Health and Primary CareOccupational, Environmental and Insurance MedicineKatholieke Universiteit LeuvenLeuvenBelgium; ^5^University Hospital LeuvenEmergency departmentKatholieke Universiteit LeuvenLeuvenBelgium; ^6^ZNA Hospital Network AntwerpAntwerpenBelgium

**Keywords:** clinical practice guidelines, evidence-based medicine, guideline development, consensus methods, human computation, games with a purpose

## Abstract

**Background:**

Guideline developers use different consensus methods to develop evidence-based clinical practice guidelines. Previous research suggests that existing guideline development techniques are subject to methodological problems and are logistically demanding. Guideline developers welcome new methods that facilitate a methodologically sound decision-making process. Systems that aggregate knowledge while participants play a game are one class of human computation applications. Researchers have already proven that these games with a purpose are effective in building common sense knowledge databases.

**Objective:**

We aimed to evaluate the feasibility of a new consensus method based on human computation techniques compared to an informal face-to-face consensus method.

**Methods:**

We set up a randomized design to study 2 different methods for guideline development within a group of advanced students completing a master of nursing and obstetrics. Students who participated in the trial were enrolled in an evidence-based health care course. We compared the Web-based method of human-based computation (HC) with an informal face-to-face consensus method (IC). We used 4 clinical scenarios of lower back pain as the subject of the consensus process. These scenarios concerned the following topics: (1) medical imaging, (2) therapeutic options, (3) drugs use, and (4) sick leave. Outcomes were expressed as the amount of group (dis)agreement and the concordance of answers with clinical evidence. We estimated within-group and between-group effect sizes by calculating Cohen’s d. We calculated within-group effect sizes as the absolute difference between the outcome value at round 3 and the baseline outcome value, divided by the pooled standard deviation. We calculated between-group effect sizes as the absolute difference between the mean change in outcome value across rounds in HC and the mean change in outcome value across rounds in IC, divided by the pooled standard deviation. We analyzed statistical significance of within-group changes between round 1 and round 3 using the Wilcoxon signed rank test. We assessed the differences between the HC and IC groups using Mann-Whitney U tests. We used a Bonferroni adjusted alpha level of .025 in all statistical tests. We performed a thematic analysis to explore participants’ arguments during group discussion. Participants completed a satisfaction survey at the end of the consensus process.

**Results:**

Of the 135 students completing a master of nursing and obstetrics, 120 participated in the experiment. We formed 8 HC groups (n=64) and 7 IC groups (n=56). The between-group comparison demonstrated that the human computation groups obtained a greater improvement in evidence scores compared to the IC groups, although the difference was not statistically significant. The between-group effect size was 0.56 (*P*=.30) for the medical imaging scenario, 0.07 (*P*=.97) for the therapeutic options scenario, and 0.89 (*P*=.11) for the drug use scenario. We found no significant differences in improvement in the degree of agreement between HC and IC groups. Between-group comparisons revealed that the HC groups showed greater improvement in degree of agreement for the medical imaging scenario (d=0.46, *P*=.37) and the drug use scenario (d=0.31, *P*=.59). Very few evidence arguments (6%) were quoted during informal group discussions.

**Conclusions:**

Overall, the use of the IC method was appropriate as long as the evidence supported participants’ beliefs or usual practice, or when the availability of the evidence was sparse. However, when some controversy about the evidence existed, the HC method outperformed the IC method. The findings of our study illustrate the importance of the choice of the consensus method in guideline development. Human computation could be an acceptable methodology for guideline development specifically for scenarios in which the evidence shows no resonance with participants’ beliefs. Future research is needed to confirm the results of this study and to establish practical significance in a controlled setting of multidisciplinary guideline panels during real-life guideline development.

## Introduction

Evidence-based clinical practice guidelines can be defined as “systematically developed statements to assist practitioner and patient decisions about appropriate health care for specific clinical conditions” [1]. They are intended to help physicians implement the burgeoning amount of scientific evidence on current medical best practices.

The development of clinical practice guidelines requires a systematic and transparent process, in which the recommendations of the clinical practice guideline (CPG) are explicitly linked to the clinical evidence. However, guidelines cannot be deduced from evidence alone, and expert opinions are needed to contextualize the evidence to the target population [2]. Furthermore, when evidence does not exist, or when there is little or incomplete evidence, the personal opinion of experts becomes more important [2]. Common methods for guideline development include the Delphi method, the nominal group technique (NGT), and the consensus development conference [3].

The high cost in time, resources, and efforts needed for the Delphi method, and the intensive commitment required for the NGT, pose important practical and logistic problems [3]. Various social-psychological influences on group discussion and decisions play an important role when face-to-face meetings or the NGT are used in the guideline development process. Previous research suggests that clinical evidence has a variable influence on guideline recommendations because of these social-psychological influences [4]. The variable influences of the clinical evidence, in turn, have an important impact on the validity and the quality of the guideline content as well as the implementation and effectiveness of the guideline [5,6].

Conducting the consensus development process entirely online is an approach that can address these concerns [7-10]. An online consensus process has the potential to involve a lot of participants and stakeholders, while offering organizational and logistic advantages in terms of cost and time savings. Social-psychological influences inherent in traditional face-to-face meetings could be eliminated by anonymously implementing the consensus process online. Explicit methods could be used to aggregate opinions.

Human-based computation is a technique in computer science in which the problems that a computer cannot yet solve are outsourced to humans. One class of human-based computation applications are the systems in which the tasks outsourced to humans are packaged as a game. These applications are called games with a purpose (GWAP). The idea behind these systems is to take advantage of people’s desire to be entertained while performing useful tasks as a side effect. This approach is effective in building large knowledge databases, but to date it has no known practical applications in medicine [11-13].

Based on the principles of successful GWAP, we developed the CPGame (clinical practice game) application as a new method for guideline development. We built a prototype based on human-based computation techniques and the goals of the experiment. Our objectives were threefold: (1) to investigate the similarities or differences in degree of agreement and evidence with an informal consensus method to explore whether the human-based computation method is a valuable alternative, (2) to investigate arguments in decision making during group discussion, and (3) to explore perceptions and opinions about the consensus method. The objectives of the study were hypothesis-generating in the first place.

## Methods

### Design

We performed a randomized controlled trial (RCT) to compare the feasibility of the human-based computation method with an informal consensus method using a face-to-face meeting. Different consensus groups participated in the trial. Each of these groups consisted of 8 participants [14,15].

We developed 4 multiple choice scenarios involving lower back pain. An example scenario is included as Multimedia Appendix 1. Participants were asked to indicate their preference between several options for the given scenario. The 4 scenarios were totally different in content and concerned the following topics: (1) medical imaging, (2) therapeutic options, (3) drug use, and (4) sick leave. The first 3 scenarios included clear levels of clinical evidence for each of the answers participants could choose from. No clinical evidence was included for the fourth scenario. The evidence for the scenarios was selected based on a previous systematic review of the Belgian Health Care Knowledge Center [16]. The evidence was graded by the Belgian Health Care Knowledge Center using the GRADE system [17]. The quality of the evidence was classified as high, moderate, low, or very low.

Students completing a master of nursing and obstetrics at the University of Leuven participated in the experiment while taking a course in evidence-based health care. All of the participants already had a bachelor of nursing and practical experience. They all had baseline knowledge about lower back pain that was sufficient to judge the clinical scenarios. They also had sufficient knowledge in evidence-based health care to understand the evidence terms and the scientific meaning of the levels of evidence. There were no exclusion criteria, and all students had Internet experience.

### Intervention and Controls

We developed the CPGame application based on human-based computation techniques. The human-based computation method is comparable to an online Delphi method packaged as a game. CPGame is a real-time collaborative application written in PHP, JavaScript, and Ajax. We used a MySQL database as the data repository. We pilot tested the technical robustness of the application with a group of trainees in family medicine.

**Figure 1:**
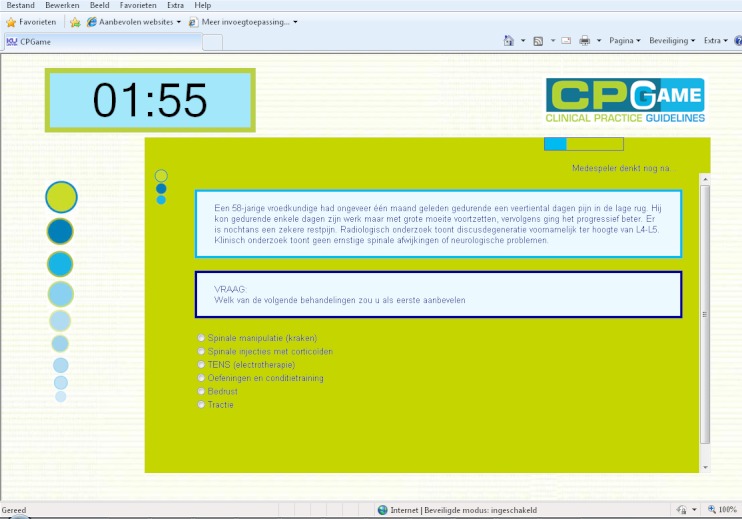
Screen capture of a multiple choice scenario in the CPGame application.

Each participant in the HC groups was anonymously paired with another participant. The 2 participants played in a team against the other teams of 2 participants (giving 4 teams of 2 students in each group). Participants were given a user ID and password to log on to the CPGame application. The 2 participants on each team were given the same multiple choice questionnaire about a clinical scenario (Figure 1).

**Figure 2:**
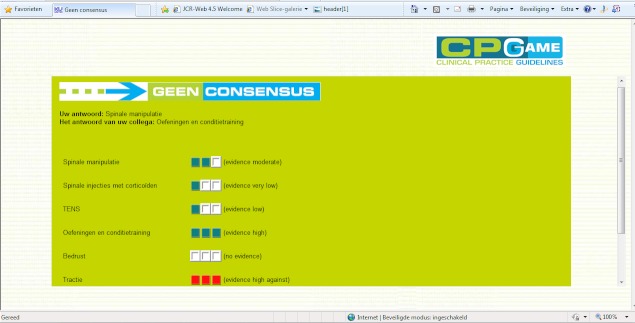
Screen capture of the information displayed on the CPGame when consensus was not reached within a team.

Independently of each other, both participants on a team gave their opinion about the case by choosing their preferred answer from the multiple choice list. When they had given their answers, the application displayed a message stating whether or not they reached consensus. If they reached consensus, they were given the next clinical scenario. If they did not reach a consensus, the application displayed the evidence for each answer and the answer of the teammate (Figure 2).

**Figure 3:**
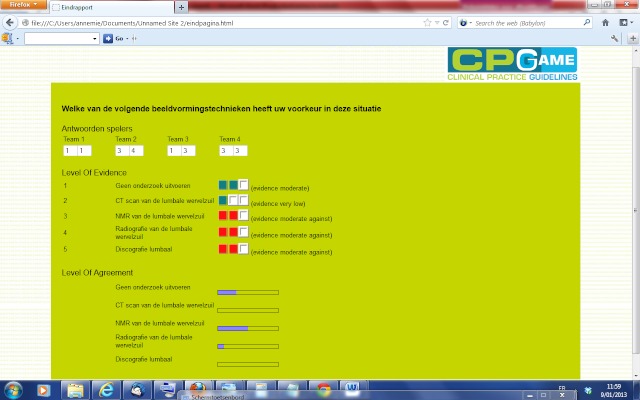
Screen capture of the final page in the CPGame application, showing the answers of all participants, the level of evidence, and the level of agreement between participants.

Each participant was given one chance to change his or her answer to try to reach consensus on a second attempt. After all the teams responded to all 4 scenarios, the CPGame application displayed the answers of all participants and the level of agreement between participants (Figure 3). Each participant reflected on his or her opinion and gave his or her final decision individually in a third round.

The game behind the human computation application consisted of a point system, a high-score list, and time pressure. These elements are described in the literature as being salient features that make GWAP fun [18]. The application determined team ranking based on the time in which a team completed all cases, the number of times consensus was reached, and the number of times the time limit was exceeded. Team ranking was only added as a competition element to make the consensus process more fun; it was not used as an outcome in final analysis. The third consensus round took place after team ranking was determined, to avoid a possible influence in the end results due to the competition element. The rules of the game were given to each participant on paper before the start of the game.

All participants completed the experiment in the same room at the same time. This one-room setting was possible because students participated in the trail during their course in evidence-based health care and had to come to the building for their class. A moderator was in the room in case there were technical problems during the experiment. The CPGame application was originally designed to be a self-directed process in which users independently participate online at their home.

The online approach to human-based computation (HC) the informal consensus method was comparable with a traditional face-to-face meeting. We took several measures to make both HC and IC methods comparable; the only difference was the mode (face-to-face versus Web-based packaged as a game).

The content of the scenarios and the evidence were similarly presented in both HC and IC groups. Before the start of the informal consensus process, each participant individually indicated his or her preference between several options for each scenario in the first round. After this first round, participants were randomly grouped into teams of 2, they were given the evidence for each treatment option, and each team discussed the cases. The discussion within the teams of 2 students was added as an additional step in the consensus process to ensure the points of measurement were equal between the 2 methods being reviewed. Each participant individually re-rated each scenario in the second round. After the second round, all 8 participants met in one group. A moderator, with experience in coordinating small groups, managed the discussion based on a written protocol designed to standardize the meetings. At the meeting, participants were told the judgments of the other group members and the research evidence. As a group, participants discussed each scenario and explored reasons for differences in opinions. After the group meeting, each participant individually re-rated each scenario in the third round.

Participants in neither the IC groups nor the HC groups were given incentives to participate in the experiment. The possible incentive of winning the game in the HC groups was neutralized by the introduction of a last consensus round outside the game.

### Randomization Procedure

All students were invited to participate in the trial a week before the experiment by one of the researchers. Participants were randomly assigned to 1 of the 2 groups (HC or IC) following simple randomization procedures. One of the researchers performed the randomization with an electronic random-list generator, initially in 1:1 ratio. A second step in the randomization procedure consisted of assigning individuals an additional consensus group number.

When they entered the computer room, participants of the human-based computation group blindly chose an envelope at random with a user ID and password to log on to the CPGame application. The envelope contained a number from 1 to 8. Numbers 1 and 2 played the game in a team, numbers 3 and 4 were a team, etc. Participants did not know each other’s numbers and did not know who would be on each team. We used the same randomization procedure in the informal consensus group after first round ratings were completed. Participants blindly chose an envelope at random with a number from 1 to 8. Predetermined pairs of numbers were used to form the teams.

Group assignments were given just before the start of the experiment. Although participants knew they were participating in a guideline development project about lower back pain, they did not know the outcomes and the goals of the project before participation. Researchers were not blinded to allocation, but outcomes were objective measures.

### Outcomes and Statistical Analyses

#### General

We conducted descriptive statistics and graphical displays to describe the sample population. Baseline data about the participants’ gender and age were compared using chi-square and Mann-Whitney U tests, as appropriate.

There were 3 points of measurement: (1) before the consensus process (round 1), (2) during the consensus process (round 2), and (3) at the end of the consensus process (round 3). Primary outcomes focused on the change of opinions towards consensus and towards evidence between round 1 and round 3. An analysis of the group’s level was warranted because of our interest in group decision making. As the group’s outcomes were treated as individual observations, we had not taken the clustering of individuals within a discussion group into account. It was appropriate to analyze the 4 scenarios separately because they were totally different in content. As such, degree of (dis)agreement and degree of evidence were calculated for each of the clinical scenarios. We used a Bonferroni adjusted alpha level of .025 (.05/2 outcome measures for each clinical scenario) for all statistical tests. Predictive Analytics SoftWare Statistics 18 was used for statistical analyses.

#### Amount of (Dis)agreement

We used a kappa statistic to express the degree of (dis)agreement within a group at the different rounds. We estimated the within-group change between round 1 and round 3 by Cohen’s d (calculated as the absolute difference between the kappa value at round 3 and the baseline kappa value at round 1, divided by the pooled standard deviation). We analyzed the statistical significance of within-group differences between round 1 and round 3 using Wilcoxon signed rank test.

We calculated between-group effect sizes for the differences in the change in agreement between the HC and IC groups to get an idea of the magnitude of the intervention effect on the amount of (dis)agreement. We calculated between-group effect sizes or Cohen’s d as the absolute difference between the mean change in agreement across rounds in HC and the mean change in agreement across rounds in IC, divided by the pooled standard deviation. We assessed differences between the HC and IC groups in the change in degree of agreement using Mann-Whitney U tests. Final kappa scores were not adjusted for their baseline values because the subjects of comparison were the differences in the change in agreement across rounds, not the differences in final agreement.

#### Amount of Concordance with Clinical Evidence

We calculated a group’s evidence score to have an idea of the degree of evidence in the answers of each group. We assigned different points to the different levels of evidence. An answer for which a high level of evidence existed got 4 points, a moderate level of evidence got 3 points, a low level of evidence got 2 points, and a very low level of evidence got 1 point. Answers for which there was evidence against got the same points with the opposite sign. Evidence points were multiplied by the number of participants who chose an answer with that level of evidence. The total sum was divided by the highest possible group’s evidence score for the specific clinical question. An evidence score of 1 meant that all group members chose the answer with the highest level of evidence.

We estimated the within-group change in evidence score between round 1 and round 3 by Cohen’s d (calculated as the absolute difference between the evidence score at round 3 and the baseline evidence score at round 1, divided by the pooled standard deviation). We analyzed the statistical significance of within-group differences between round 1 and round 3 using Wilcoxon signed rank test.

We calculated the between-group effect sizes for the differences in the change in evidence score between the HC and IC groups. We calculated the between-group effect sizes or Cohen’s d as the absolute difference between the mean change in evidence scores across rounds in HC and the mean change in evidence scores across rounds in IC, divided by the pooled standard deviation. We assessed differences between the HC and IC groups in the change in evidence score using Mann-Whitney U tests.

#### Thematic Analysis

We used a hidden camera to record meetings of the informal consensus groups to explore arguments in each group’s decision-making process. Hiding the camera was necessary to avoid social-psychological influences that arise with the awareness of recording. Two analysts anonymously transcribed and independently coded the recorded meetings of the face-to-face groups. Each communicative function within an utterance was defined as a dialogue act. Each dialogue act was coded and classified under a theme. We developed a preliminary list of themes based on the published list of themes created by Gardner et al [4]. We applied this preliminary list to the transcripts and adapted it to the specific situation of our populations.

We used the length of discussion time as a process measure of the group discussion. Discussion time was defined as the elapsed time between the start and the end of a group’s decision-making activities.

We gave the students a paper questionnaire after the consensus process to explore perceptions about the consensus method. After the experiment, participants in the informal consensus group were notified about the hidden camera. Offline, we obtained informed consent to use the results for analysis from all participants. If one of the participants did not agree to allow us to use the hidden camera footage, we did not use the recordings and results of that group.

We obtained approval from the University Hospitals Leuven Medical Ethics Committee for this study in December 2009. The full protocol and the approval form can be obtained from the corresponding author.

## Results

**Figure 4:**
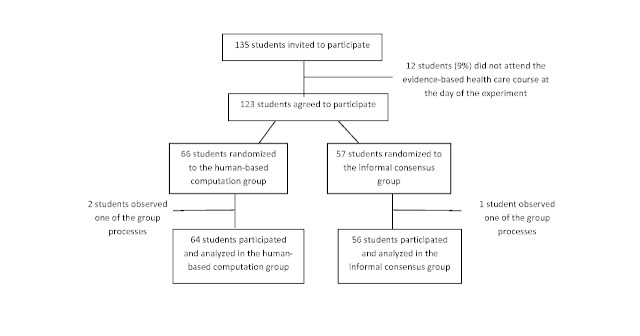
Flowchart showing participants in the trial.

A total of 120 out of 135 students completing a master of nursing and obstetrics participated in the experiment. The participants formed 8 HC and 7 IC groups. Fewer students than expected attended the experiment, so only 7 instead of 8 informal consensus groups could be constituted. A total of 3 students were not assigned to groups. They participated as observers of the group’s process and were not included in analysis. All participants who were randomly assigned to a group were analyzed in their original assigned groups (Figure 4).

The 2 groups were similar in terms of age and gender. There were no statistically significant differences in baseline evidence score and baseline agreement at round 1 (Table 1).

**Table 1:** Baseline demographic data and outcome scores.

		Human-based computation (8 groups, 64 participants)	Informal consensus (7 groups, 56 participants)
Year of birth		1986	1986
n (%) female		56 (88%)	50 (89%)
**Evidence score**			
	Medical imaging (95% CI)	-0.15 (-0.40 to 0.10)	-0.14 (-0.32 to 0.04)
	Therapeutic options (95% CI)	0.67 (0.51 to 0.82)	0.81 (0.72 to 0.89)
	Drug use (95% CI)	0.43 (0.31 to 0.55)	0.49 (0.36 to 0.63)
**Degree of agreement**			
	Medical imaging (95% CI)	0.29 (0.25 to 0.32)	0.29 (0.22 to 0.36)
	Therapeutic options (95% CI)	0.45 (0.27 to 0.62)	0.5 (0.31 to 0.69)
	Drug use (95% CI)	0.21 (0.11 to 0.31)	0.25 (0.11 to 0.38)
	Sick leave (95% CI)	0.52 (0.37 to 0.66)	0.60 (0.34 to 0.85)

### Amount of (Dis)agreement

**Figure 5:**
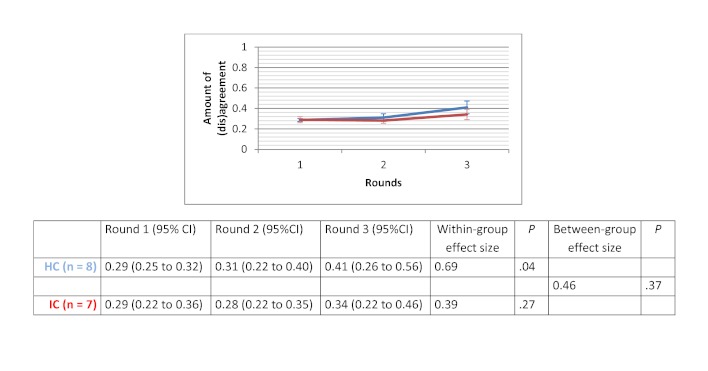
Amount of (dis)agreement for the medical imaging scenario.

**Figure 6:**
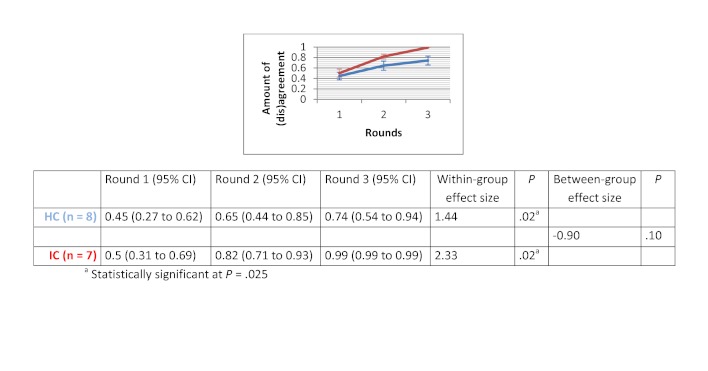
Amount of (dis)agreement for the therapeutic options scenario.

**Figure 7:**
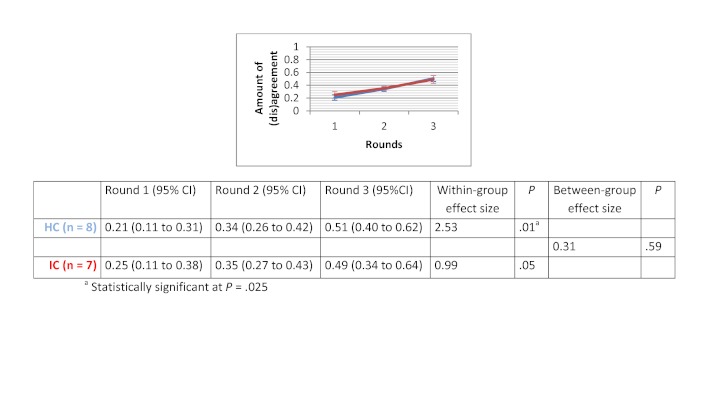
Amount of (dis)agreement for the drug use scenario.

**Figure 8:**
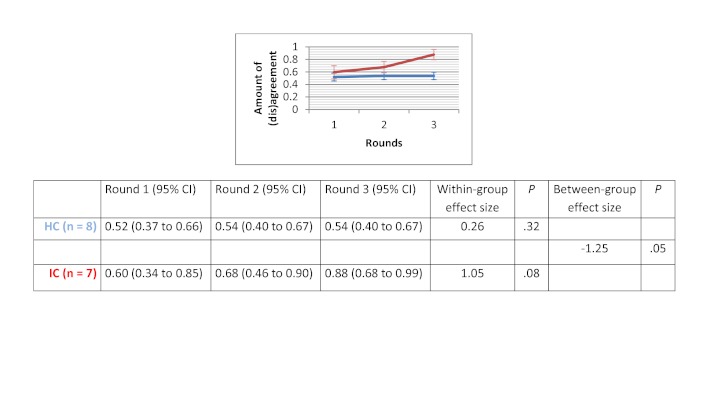
Amount of (dis)agreement for the sick leave scenario.

Within-group effect sizes (Cohen’s d) varied between 0.26 and 2.53 in the HC groups and were statistically significant for the therapeutic options scenario (d=1.44 with *P*=.02) and for the drug use scenario (d=2.53 with *P*=.01). Within-group effect sizes in the IC groups varied between 0.39 and 2.33. IC groups showed a significant improvement in the degree of agreement for the therapeutic options scenario (d=2.33 with *P*=.02). No significant differences in improvement of degree of agreement were found between HC and IC groups. Between-group comparisons revealed that the HC groups showed greater improvement in degree of agreement for the medical imaging scenario (d=0.46 with *P*=.37) and the drug use scenario (d=0.31 with *P*=.59). The opposite was true for the therapeutic options scenario (d=-0.9 with *P*=.10) and the sick leave scenario (d=-1.25 with *P*=.05). The change in degree of agreement across the 3 rounds, within-group effect sizes, and between-group effect sizes are displayed in Figures 5 to 8.

### Concordance with Clinical Evidence

**Figure 9:**
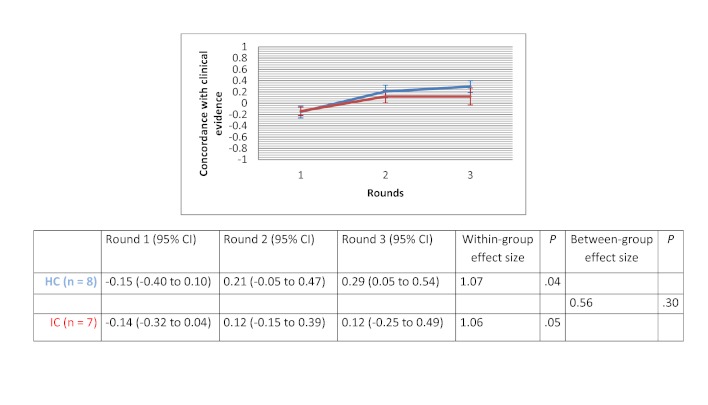
Concordance with clinical evidence for the medical imaging scenario.

**Figure 10:**
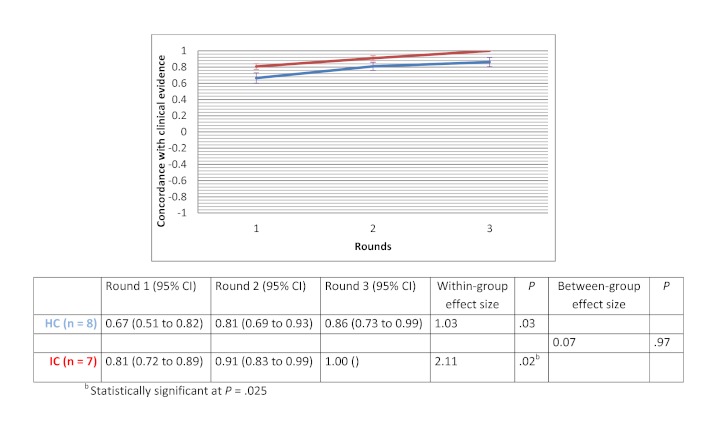
Concordance with clinical evidence for the therapeutic options scenario.

**Figure 11:**
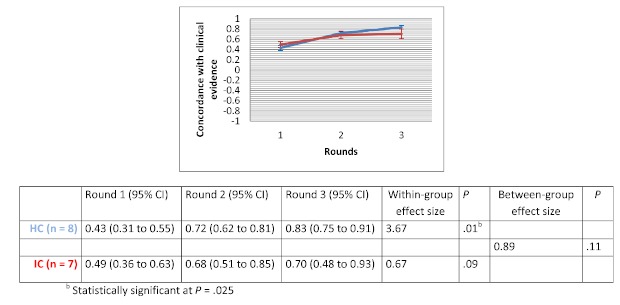
Concordance with clinical evidence for the drug use scenario.

After 3 rounds, the mean evidence score increased for all clinical scenarios in both groups. Within-group changes showed a significant improvement in evidence score for the drug use scenario in the HC groups (d=3.67 with *P*=.01) and for the therapeutic options scenario in the IC groups (d=2.11 with *P*=.02). The between-group comparison demonstrated that the human-based computation groups obtained a greater improvement in evidence scores compared to the IC groups, although the difference was not statistically significant. Between-group effect size was 0.56 (*P*=.30) for the medical imaging scenario, 0.07 (*P*=.97) for the therapeutic options scenario and 0.89 (*P*=.11) for the drug use scenario. Figures 9 to 11 show the change in mean group’s evidence score across the 3 rounds, within-group effect sizes and between-group effect sizes for the different clinical scenarios.

### Thematic Analysis

**Figure 12:**
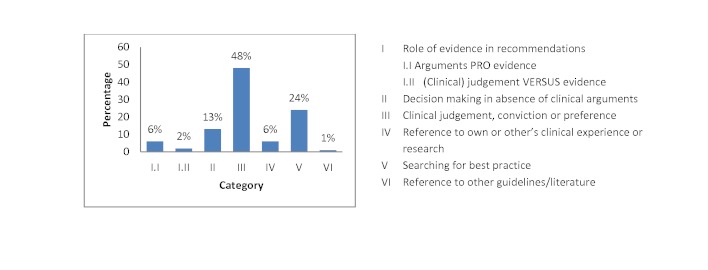
Percentage of arguments in the different categories of the coding scheme.

Figure 12 shows the frequencies with which the different themes appeared across the meetings. The results show a greater focus on clinical preference than on clinical evidence. Themes relating to clinical judgment or preference occurred the most (177/369, 48% arguments), while there were relatively few arguments explicitly pro evidence (23/369, 6% arguments). Group discussions were characterized by a high degree of uncertainty, which is shown by the high percentages (87/369, 24%) in category V, and few references to own or other’s clinical experiences (23/369, 6%). Only 5 out of 369 agreements (1%) could be classified under the category “reference to other guidelines or literature.” All individual meetings followed more or less the same patterns in themes. A mean Cohen’s kappa of 0.77 was reached for interanalyst agreement.

Mean discussion time for the 4 clinical scenarios was 32.9 minutes (± 6.5 minutes) in the IC groups and 14.6 minutes (± 2.2 minutes) in the HC groups. Analysis of the time intervals revealed a statistically significant shorter discussion time in the HC groups compared to the IC groups (*P=*.001). Participant satisfaction scores are shown in Table 2 and 3.

**Table 2:** Satisfaction scores.

		Human-based computation method (HC) n (%)	Informal consensus method (IC) n (%)
I am satisfied with the group answer	Strongly agree	7 (11)	12 (21)
	Agree	30 (48)	30 (53)
	Undecided	21 (33)	11 (20)
	Disagree	5 (8)	2 (4)
	Strongly disagree	0 (0)	1 (2)
		63 (100)	56 (100)
I find the levels of evidence important when making my decision	Strongly agree	32 (50)	18 (32)
	Agree	25 (39)	32 (57)
	Undecided	6 (9)	5 (9)
	Disagree	1 (1)	1 (2)
	Strongly disagree	0 (0)	0 (0)
		64 (100)	56 (100)

**Table 3:** Decision-making scores.

I would describe the decision-making process as:						
Efficient	1 n (%)	2 n (%)	3 n (%)	4 n (%)	5 n (%)	Not efficient
HC	6 (10)	30 (48)	14 (23)	12 (19)	0 (0)	
IC	11 (20)	27 (48)	15 (27)	1 (2)	2 (4)	

## Discussion

### Principal Results

For the cases with evidence, changes in answers across rounds were more evidence-based in the HC groups compared to the IC groups. HC groups obtained a greater improvement in evidence scores compared to the IC groups. The anonymity of the participants in the HC game evidently avoided direct social-psychological influencing, as intended.

Differences in the improvement in agreement across rounds were better in the HC groups for the medical imaging scenario and the drug use scenario, but not for the therapeutic options scenario. The evidence score for that scenario was already relatively high starting at round 1 in the IC groups. The evidence supported students’ beliefs, values, and preexisting opinions and little group pressure was needed to convince a few individuals to reach full consensus in the IC groups.

For the sick leave scenario, which did not include clinical evidence, the informal consensus (IC) groups demonstrated closer group agreement compared to the human computation (HC) groups. Opinions were more likely to shift when groups met face-to-face, as suggested by the study of Hutchings et al [19]. The choice of the degree of (dis)agreement as a process measure assumed that consensus is a good outcome and that IC groups fared better for the scenario without evidence. Many guideline developers would disagree with the fact that consensus is a good outcome. Although we acknowledge this point of view, we believe it was appropriate to use kappa values as a process measure because reaching consensus is the primary goal of each consensus process.

Supplying the evidence at round 2 had an influence on group judgment (shown by the positive within-group Cohen’s d for the evidence score) as well in the HC groups as in the IC groups. However, thematic analysis in the IC groups revealed that choices were more likely to be based on clinical judgment or conviction, rather than on clinical evidence (as supported by Raine [20]). The few evidence arguments in the IC groups (6%) were in sharp contrast with the results of the questionnaire, where 89% of the participants in the IC groups perceived the influence of the evidence as important. Perceptions did not correspond with arguments used in practice.

Hutching et al [19] demonstrated earlier that direct exposure to arguments and (dominating) personalities could lead guideline development groups in different directions. The anonymity of the participants in the HC groups eliminated important aspects of social-psychological influences, which gained the upper hand in the IC groups. It is surprising that so little evidence arguments were quoted in the IC groups. These findings could confirm the idea of Sauerland et al [21] that evidence in IC groups is used to confirm preexisting opinions, rather than to change them.

### Limitations of the Study

There were some methodological and practical limitations to our study. The limited number of clinical scenarios, especially for the type of case without evidence, may reduce the generalization of the results. It was a proof-of-concept hypothesis-generating study, so we did not power the study before the start. The obtained power was not robust enough for a reliable detection of a between-group effect, which increased our chance of false-negative conclusions concerning statistical significance.

The high degree of variability between the individual groups may be seen as realistic reflections of variations in clinical perspective. However, it also confirms the importance of the composition of the guideline panel and the choice of the moderator [22-25]. What is to be decided is often already determined with the selection of the deciders [26]. The effect of the panel composition should be minimal because it concerned a rather homogeneous population (students in an evidence-based heath care course) randomized to the different groups (HC or IC). Although in contrast with real multidisciplinary guideline panels, we chose a rather homogeneous group of students with limited expertise as the subject of the experiment to partly control for the social-psychological influences rising from multistatus groups. The choice of the study population was appropriate based on the research, which explored the influence of the consensus method on the change of opinions towards consensus and evidence, rather than the content of consensus. We decided that a high degree of lower back pain specialization was not necessary in this preliminary phase of the research. Because of the exploratory and early phase nature of the work, the choice of these participants with baseline knowledge about lower back pain was justified. We stressed spontaneous group interaction, rather than reaching consensus, to minimize the influence of the moderator.

Time intervals did not represent real discussion times in multidisciplinary guideline panels because of the aforementioned differences between our discussion groups and these expert panels. However, time analysis demonstrated the potential of the human-based computation method to be an efficient consensus method. The thematic analyses may also differ from discussions in expert groups or other guideline development groups. Participants were not specialized in one medical domain and did not have the intention to over-state the effectiveness of their specialist intervention, which may have reduced the amount of contentious issues. The thematic analyses also reflected the attitude towards clinical practice of a new generation of professionals recently educated in evidence-based health care.

We gave equal weighting to the different levels of evidence in our calculation of the evidence score. We are aware that not all people give equal weights to a difference between evidence low and evidence moderate, or evidence moderate and evidence high, etc. However, the literature did not provide a scientific basis to assign unequal weights to the different evidence levels. As a consequence, equal weighting of the different evidence levels was considered as appropriate as unequal weighting.

Another limitation of the study is the lack of a third online Delphi group without a game component to allow us to separately study the effect of the online approach and the effect of the game itself. While eliminating social-psychological influences inherent in face-to-face groups, we introduced a new psychological element of competition in the HC groups due to the game component. This could have influenced the results at round 2, but did not affect our final results. Participants got the chance to reconsider their answers in a third individual round after finishing the game.

The students participated in the experiment during the hours of their class in evidence-based health care. Because of this, we could easily conduct the experiment with all the participants in one room. No additional logistic facilities were required; students had to come to the building for their class. This is in contrast to the suggested advantages of an online process, where experts participate at home. However, the method was originally designed to be a self-directed process, participants worked individually on a computer during the experiment, and no verbal communication with the moderator took place. Therefore, we believe the results could be easily generalized to a real-world online process.

### Relation to Other Studies

Relatively few studies compared different consensus methods for guideline development. Many of them differed in the consensus methods they compared or in the way they operationalized the method. Washington et al [9] and Kadam et al [22] did not demonstrate differences in final ratings between the consensus methods. Shekelle et al found limited differences in their study [27]. Hutching et al [19] showed greater within-group agreement in nominal groups compared with Delphi groups, which was contrary to the earlier research of Leape et al [28]. The systematic review of Murphy et al [3] concludes that formal methods generally perform better than informal ones and may be better for consensus development.

Our newly developed method of guideline development by human-based computation proved very useful in the introduction of clinical evidence arguments, while neutralizing for social-psychological influences by authoritarian opinions.

The findings of our study illustrate once more the importance of the choice of the consensus method in guideline development. Giving the same evidence summary and using a consensus process, HC and IC groups could come to different group views. The influence of the consensus method seemed to depend on the type of clinical question. Overall, the use of the informal consensus method may be appropriate as long as the evidence supports participants’ beliefs or usual practice, or when the availability of the evidence is sparse. However, when some controversy about the evidence exists, one could doubt the appropriateness of the informal consensus method. Because guideline programs are intended to reduce inappropriate variations in health care, guidelines are more important for clinical questions where the evidence shows no resonance with participants’ beliefs. Human computation outperformed the informal consensus method for this type of clinical questions.

### Human Computation: an Acceptable Method for Guideline Development?

Participants perceived the human-based computation method as pleasant and enjoyable. Satisfaction was similar in the HC and IC groups. Only 3 out of 56 participants (5%) were dissatisfied with the group answer in the IC groups, while 5 out of 63 participants (8%) were dissatisfied in the HC groups. This is in contrast to the literature on computer-mediated communication versus face-to-face groups [10,29], where lower satisfaction is reported in computer groups in general. The additional game component in the computer groups could be a possible explanation for these higher satisfaction levels.

However, the major strengths of the method (the anonymity of panelists, the elimination of social-psychological influences in face-to-face meetings, and the possibility to participate in an online development group from a distance) was at the same time a reason for lower satisfaction. Participants perceived lack of group discussion and interaction in the HC groups as a negative aspect of the method. Participants seemed to need the opportunity to find out reasons for other members’ decisions [3]. Although the human-based computation method has the potential to offer advantages in terms of logistics and more objective decision making, participants perceived the method’s efficiency more negatively in the human-based computation group than in the informal consensus group. Of the 62 in the HC groups participants, 12 (19%) were dissatisfied with the efficiency of the HC method, while 3 out of 56 participants in the IC groups (5%) were dissatisfied with the efficiency of the informal consensus method. This was probably related to the lack of understanding of other participants’ arguments.

The current format of the CPGame application was built for the purposes of the experiment. This prototype was essential to test the feasibility and the acceptability of the specific method for guideline development. However, if it is to be useful in practice, a more complex application will be needed.

We believe it is important to draw on the advantages of both methods (human-based computation and face-to-face meetings) in view of future system improvements. The exploration of group views should be incorporated, while maintaining the existing advantages of human-based computation. A hybrid method could be considered, including an extra button to ask for the arguments of other players to complement the human computation method. An extension to an asynchronous mode would also allow large-scale advantages and let people choose when they participate in the process. We chose the current format of multiple choice questions to test the feasibility of the method because of its plainness. Extensions to other question formats are also feasible and probably more adapted to guideline development.

### Conclusions

The findings of our study illustrate the importance of the choice of the consensus method in guideline development. Giving the same evidence summary and using a different consensus process, two groups can come to different group views, which implies a considerable risk towards conflicting guideline recommendations on the same topic.

Human computation could be a time efficient and acceptable methodology for guideline development specifically for scenarios in which the evidence shows no resonance with participants’ beliefs. Changes in evidence scores and agreement after 3 rounds were higher in HC groups compared to IC groups for this type of scenario. Controlled feedback is given while eliminating the social-psychological components of a group process. Level of evidence and level of agreement are separated, which could increase transparency of the guideline-development process.

Future research is needed to confirm the results and to establish practical significance in a controlled setting of multidisciplinary guideline panels during real-life guideline development.

## Multimedia Appendix 1

Example of a multiple-choice questionnaire based on a clinical scenario involving lower back pain.

## Multimedia Appendix 2

CONSORT E-health checklist V1.6.1 [30].

## Abbreviations

CPG: clinical practice guideline
CPGame: clinical practice game
GWAP: games with a purpose
HC: human-based computation
IC: informal face-to-face consensus method
NGT: nominal group technique
RCT: randomized controlled trial
