# Supplementary material for: Human Computation as a New Method for Evidence-Based Knowledge Transfer in Web-Based Guideline Development Groups: Proof of Concept Randomized Controlled Trial
Source: J Med Internet Res. 2013 Jan 17;15(1):e8. doi: 10.2196/jmir.2055 (PMC3636290; doi:10.2196/jmir.2055)
Supplement: Supplementary file 1 [file jmir_v15i1e8_app1.pdf]

Example of clinical scenario presented as multiple choice question

A 36-year-old female geriatric nurse presented with pain of her lower back that radiates bilaterally on the back of the thigh to the knee. The patient's pain began one months ago since turning a obese bed patient. She had been able to control similar problems previously with several self help measures. Conservative treatment with pain relief did not provide adequate response after 10 weeks. The patient starts worrying and keeps on asking for a radiological examination to be reassured. Your opinion is asked.

Which of the following radiological examinations do you prefer in this situation?

❍ A radiological examination is not necessary	evidence moderate
❍ CT scan	evidence very low
❍ MRI scan	evidence moderate against
❍ X-rays	evidence moderate against
❍ Discography	evidence moderate against

Quality of evidence and definitions according GRADE

- *Evidence high* — Further research is very unlikely to change our confidence in the estimate of effect
- *Evidence moderate* — Further research is likely to have an important impact on our confidence in the estimate of effect and may change the estimate
- *Evidence low* — Further research is very likely to have an important impact on our confidence in the estimate of effect and is likely to change the estimate
- *Evidence very low* — Any estimate of effect is very uncertain

Reference: Guyatt GH, Oxman AD, Vist G, Kunz R, Falck-Ytter Y, Alonso-Coello P, et al. Rating quality of evidence and strength of recommendations GRADE: an emerging consensus on rating quality of evidence and strength of recommendations. BMJ 2008;336:924-926. PMID: 18436948.
