# Supplementary material for: Human Computation as a New Method for Evidence-Based Knowledge Transfer in Web-Based Guideline Development Groups: Proof of Concept Randomized Controlled Trial
Source: J Med Internet Res. 2013 Jan 17;15(1):e8. doi: 10.2196/jmir.2055 (PMC3636290; doi:10.2196/jmir.2055)
Supplement: Supplementary file 2 [file jmir_v15i1e8_app2.pdf]

CONSORT-EHEALTH Checklist V1.6 Report

Manuscript Number 2055

Date completed 8/25/2011 14:50:29

by Annemie Heselmans

Human computation as a new method for evidence-based knowledge transfer in web-based guideline development groups: A proof of concept randomized controlled trial.

TITLE

1a-i) Identify the mode of delivery in the title

"web-based guideline development groups"

1a-ii) Non-web-based components or important co-interventions in title

not applicable

1a-iii) Primary condition or target group in the title

not applicable, the paper is related to a new method for guideline development in general

ABSTRACT

1b-i) Key features/functionalities/components of the intervention and comparator in the METHODS section of the ABSTRACT

"New methods are welcomed that facilitate a methodological sound decision making process. One class of human computation applications are the systems that aggregate knowledge while playing a game (Games With A Purpose). Games With A Purpose have already been proven to be effective in building common sense knowledge databases."

"The web-based method of human computation (HC) was compared with an informal face-to-face consensus method (IC)."

1b-ii) Level of human involvement in the METHODS section of the ABSTRACT

Not applicable, the internet is used as technology to support a consensus method for guideline development. Involvement of experts is inherent in guideline development.

1b-iii) Open vs. closed, web-based (self-assessment) vs. face-to-face assessments in the METHODS section of the ABSTRACT

"A randomized design was set up to study two different methods for guideline development within a group of advanced students following a master of nursing and obstetrics. Students participated in the trial during their course of Evidence-Based Health Care. "

"The web-based method of human computation (HC) was compared with an informal face-to-face consensus method (IC)."

"Outcomes were expressed as the amount of group (dis)agreement and the concordance of answers with clinical evidence."

"A thematic analysis was performed to explore participants' arguments during group discussion. A satisfaction survey was administered at the end of the consensus process."

1b-iv) RESULTS section in abstract must contain use data

"120 (out of 135) students following a master of nursing and obstetrics participated in the experiment. 8 HC groups (n=64) and 7 IC groups (n=56) were formed. The between-group comparison demonstrated that the human computation groups obtained a greater improvement in evidence scores compared to the IC groups, although not statistically significant. Between-group effect size was 0.56 (p=.30) for the clinical scenario of medical imaging, 0.07 (p=.97) for the clinical scenario of therapeutic options and 0.89 (p=.11) for the scenario of drug use. No significant differences in improvement in degree of agreement were found between HC and IC groups. Between-group comparisons showed that the HC groups showed greater improvement in degree of agreement for the scenario of medical imaging (Cohen's d=0.46 with p=.37) and the scenario of drug use (Cohen's d=0.31 with p=.59). Very few evidence arguments (6%) were quoted during informal group discussions."

1b-v) CONCLUSIONS/DISCUSSION in abstract for negative trials

Not applicable

INTRODUCTION

2a-i) Problem and the type of system/solution

"The high cost in time, resources and efforts needed for the Delphi procedure and the intensive commitment required for the NGT poses important practical problems. There are various social-psychological influences on group discussion and decisions that play an important role in the guideline development process of a face-to-face meeting or a NGT. Previous research suggests that clinical evidence has a variable influence on guideline recommendations because of these social-psychological influences. This in turn has an important impact on the validity and the quality of the guideline content, the implementation and effectiveness of the guideline."

"A method for addressing these concerns is to conduct the process entirely online. An online consensus process could have the potential to involve a lot of participants and stakeholders while offering organizational and logistic advantages in terms of cost and time savings. Social psychological influences inherent in traditional face-to-face meetings could be eliminated by the online possibilities to anonymously implement the consensus process. Explicit methods could be used to aggregate opinions."

"Based on the principles of the success of multiplayer online games (or Games With A Purpose) we developed an application 'CPGame' as a new method for guideline development"

2a-ii) Scientific background, rationale: What is known about the (type of) system

As aforementioned, human computation was chosen as a method to address existing methodological and practical concerns in guideline development. The method has already been proven to be effective in building large knowledge databases but has no practical applications in medicine to date.

METHODS

3a) CONSORT

"Objectives were threefold. First, to investigate the similarities or differences in degree of agreement and evidence with an informal consensus method in order to explore whether the method of human computation is a valuable alternative. Second, to investigate arguments in decision making during group discussion. Third, to explore perceptions and opinions about the consensus method. The objectives of the study were hypothesis-generating in the first place."

3b-i) Bug fixes, Downtimes, Content Changes

Not applicable

4a-i) Computer / Internet literacy

"There were no exclusion criteria, all students had internet experiences."

4a-ii) Open vs. closed, web-based vs. face-to-face assessments:

"A randomized controlled trial was performed to compare the method of human computation with an informal consensus method using a face to face meeting. "

"Students following a master of nursing and obstetrics at the University of Leuven participated in the experiment during their course of evidence-based health care. "

"Participants got a user ID and password to log on to the computer application. "

"Outcomes were expressed as the amount of group (dis)agreement and the concordance of answers with clinical evidence. "

"Meetings of the informal consensus groups were recorded with a hidden camera to explore arguments in group's decision making. The recorded meetings of the face-to-face groups were anonymously transcribed and independently coded by two analysts. "

"A questionnaire on paper was administered to the students after the consensus process to explore perceptions about the consensus method. "

4a-iii) Information giving during recruitment

"Details of the allocated group were given just before the start of the experiment. Whereas participants knew they were participating in a guideline development project about low back pain, they did not know the outcomes and the goals of the project before participation. Researchers were not blinded to allocation but outcomes were objective measures."

"Informed consent to use the results for analysis was obtained offline from all participants."

4b-i) Report if outcomes were (self-)assessed through online questionnaires

Primary outcomes were objective measures.

4b-ii) Report how institutional affiliations are displayed

Not applicable

5-i) Mention names, credential, affiliations of the developers, sponsors, and owners

"The authors and evaluators are the developers of the human computation application"
5-ii) Describe the history/development process
"Technical robustness of the application was pilot tested in a group of trainees in family medicine."

The manuscript describes the first evaluation of a prototype, no other formal evaluations were done..
5-iii) Revisions and updating
Not applicable
5-iv) Quality assurance methods
"The evidence for the scenarios was selected based on a previous systematic review of the Belgian Health Care Knowledge Centre. The Belgian Health Care Knowledge Centre uses a standardized protocol and methodology to perform their systematic reviews"
5-v) Ensure replicability by publishing the source code, and/or providing screenshots/screen-capture video, and/or providing flowcharts of the algorithms used
Three screenshots are added as figures in the manuscript.
5-vi) Digital preservation
The paper does not currently address this item. It is a real-time collaborative application based on an interaction between players. A demo has not been developed at the moment.
5-vii) Access
"Students following a master of nursing and obstetrics at the University of Leuven participated in the experiment during their course of evidence-based health care. "

"Participants got a user ID and password to log on to the computer application. "

A demo for individual use is currently not available, screenshots are incluced in the manuscript.
5-viii) Mode of delivery, features/functionalities/components of the intervention and comparator, and the theoretical framework
Components and features of the intervention and control groups are described in detail in the Methods section of the manuscript.
5-ix) Describe use parameters
Not applicable, the evaluation of the intervention took place during a regular curricular course of the students.
5-x) Clarify the level of human involvement
The level of human involvement was described in the Methods section. Students participated during their course of evidence-based health care. The rules of the game were individually presented on paper before the start of the game. A moderator was in the room in case of technical problems.

Rules of the game could be presented online in case of a routine application outside of a RCT setting. This manual could be supplemented with a demo version of the software.
5-xi) Report any prompts/reminders used
Not applicable
5-xii) Describe any co-interventions (incl. training/support)
There were no co-interventions accompanying the human computation application.
6a-i) Online questionnaires: describe if they were validated for online use and apply CHERRIES items to describe how the questionnaires were designed/deployed
No online questionnaires were used.
6a-ii) Describe whether and how "use" (including intensity of use/dosage) was defined/measured/monitored
Not applicable, 'use' of the application was not relevant in view of the study objectives.
6a-iii) Describe whether, how, and when qualitative feedback from participants was obtained
"A questionnaire on paper was administered to the students after the consensus process to explore perceptions about the consensus method"
7a-i) Describe whether and how expected attrition was taken into account when calculating the sample size
"It was a proof of concept hypothesis-generating study so we did not power the study before the start. "
7b) CONSORT
No interim analyses were done in this feasibility study.
8a) CONSORT

"Participants were randomly assigned to one of the two groups (HC or IC) following simple randomization procedures. Randomization was performed by the researcher with an electronic random list generator, initially in 1:1 ratio. A second step in the randomization procedure consisted of the individual assignment of an additional consensus group number.

When entering the building, participants of the human computation group blindly chose an envelope at random with a user ID and password to log on to the computer application (CPGame). The envelope contained a number from one to eight. Number 1 and number 2 played the game in one team, number 3 together with number 4, etc. Participants did not know the numbers of each other and did not know the person to who they played. The same randomization procedure was used in the informal consensus group after completion of first round ratings. Participants blindly chose an envelope at random with a number from one to eight. Predetermined couples of numbers formed the teams.

Details of the allocated group were given just before the start of the experiment."

8b) CONSORT

See item 8a

9) CONSORT

See item 8a

10) CONSORT

Participants were enrolled and assigned to the interventions by one of the researchers. Randomization was performed by one of the researchers with an electronic random list generator.

11a-i) Specify who was blinded, and who wasn't

"Researchers were not blinded to allocation but outcomes were objective measures."

11a-ii) Discuss e.g., whether participants knew which intervention was the "intervention of interest" and which one was the "comparator"

"Whereas participants knew they were participating in a guideline development project about low back pain, they did not know the outcomes and the goals of the project before participation. "

11b) CONSORT

"The informal consensus method was comparable with a traditional face-to-face meeting. Several measures were taken to make both HC and IC methods comparable, the only difference was the mode (face-to-face versus web-based packaged as a game). The content of the scenarios and the evidence were similarly presented in both HC and IC groups."

"The discussion within groups of two students was added as an additional step in the consensus process to become equivalence in the points of measurement between the two methods. "

12a) CONSORT

See item 6a

"As group's outcomes were treated as individual observations, the clustering of individuals within a discussion group had not to be taken into account. "

12a-i) Imputation techniques to deal with attrition / missing values

There were no missing data for the primary outcomes.

12b) CONSORT

Subgroup analyses were not done but an additional thematic analysis was done in the informal consensus groups.

"Meetings of the informal consensus groups were recorded with a hidden camera to explore arguments in group's decision making. The recorded meetings of the face-to-face groups were anonymously transcribed and independently coded by two analysts. Each communicative function within an utterance was defined as a dialogue act. Each dialogue act was coded and classified under a theme. A preliminary list of themes was drawn upon the published list of themes of Gardner et al. This preliminary list was applied to the transcripts and adapted to the specific situation of our populations."

RESULTS

13a) CONSORT

See flowchart figure 4.

"A total of 120 out of 135 students following a master of nursing and obstetrics participated in the experiment. 8 human computation groups (HC) and 7 informal consensus groups (IC) were formed. At the moment of the experiment, less students attended than expected and only 7 instead of 8 informal consensus groups could be constituted. A total of 3 students remained. They participated as observator of the group's process but were not included in analysis. All participants who were randomly assigned were analysed in their original assigned groups "

13b) CONSORT
See item 13a
13b-i) Attrition diagram
See flowchart figure 4
14a) CONSORT
Dates defining the periods of recruitment and follow-up were not relevant, recruitment and follow-up took place at the same moment.
14a-i) Indicate if critical “secular events” fell into the study period
No secular events fell into the study period.
14b) CONSORT
The trial was not early stopped.
15) CONSORT
Available at table 1.
15-i) Report demographics associated with digital divide issues
It concerned a rather homogeneous group of master students with almost no digital divide issues.
16-i) Report multiple “denominators” and provide definitions
Definitions of the denominators were provided in the Methods section. See item 6a.
16-ii) Primary analysis should be intent-to-treat
"All participants who were randomly assigned were analysed in their original assigned groups "
17a) CONSORT
Table 2 and 3 provide within-group and between-group effect sizes with 95%CI's. These results are also described in the text.
17a-i) Presentation of process outcomes such as metrics of use and intensity of use
Use and intensity of use were not part of the study objectives.
17b) CONSORT
No binary outcomes were studied.
18) CONSORT
Thematic analyses of the informal consnsus groups were reported as additional paragraph in the Results section and corresponding figure.
18-i) Subgroup analysis of comparing only users
A subgroup analysis of comparing only users was not done.
19) CONSORT
Nor relevant in a trial for guideline development.
19-i) Include privacy breaches, technical problems
No technical problems or unintended incidents occured during the experiment.
19-ii) Include qualitative feedback from participants or observations from staff/researchers
Three students participated as observator of the group’s process but were not included in analysis. These qualitative analyses were neither reported in the text.

Qualitative feedback of the participants themselves were used in the Discussion section as background for the interpretation of results.

DISCUSSION

20-i) Typical limitations in ehealth trials

The generalization of the thematic analysis may be reduced by the choice of the study population. The thematic analyses may be different from discussions in expert groups or other guideline development groups. Participants were not specialized in one medical domain and did not have the intention to over-state the effectiveness of their specialist intervention which may have reduced the amount of contentious issues. On the other side, the thematic analyses reflected the attitude towards clinical practice of a new generation of professionals recently educated in evidence-based health care.

Equal weighting was given to the different levels of evidence in the calculation of the evidence score. We are aware that not all people give equal weights to a difference between evidence low and evidence moderate or evidence moderate and evidence high, etc. However, a scientific base to assign unequal weights to the different evidence levels was not available in the literature. As a consequence, equal weighting of the different evidence levels was considered as appropriate as unequal weighting.

The choice of the degree of (dis)agreement as a process measure assumed that consensus is a good outcome. Although this is not always true, we believe it was appropriate to use Kappa values as a process measure as reaching consensus is the primary goal of each consensus process.

Another limitation of the study is the lack of a third online Delphi group without game component to allow us to separately study the effect of the online approach and the effect of the game itself. While eliminating social psychological influences inherent in face-to-face groups, we introduced a new psychological element of competition in the HC groups due to the game component. This could have had an influence on the results at round 2 but did not affect our final results. Participants got the chance to reconsider their answers in a third individual round after finishing the game."

21-i) Generalizability to other populations

See item 20

21-ii) Discuss if there were elements in the RCT that would be different in a routine application setting

See item 20

22-i) Restate study questions and summarize the answers suggested by the data, starting with primary outcomes and process outcomes (use)

"For the cases with evidence, changes in answers across rounds were more evidence-based in the HC groups compared to the IC groups. HC groups obtained a greater improvement in evidence scores compared to the IC groups. The anonymity of the participants to the HC game evidently avoided direct psychosocial influencing, as intended.

Differences in the improvement in agreement across rounds were in the advantage of the HC groups for the scenario of medical imaging and the scenario of drug use, but not for the scenario of the therapeutic options. The evidence score for that scenario was already relatively high starting at round 1 in the IC groups. The evidence supported students' beliefs, values and pre-existing opinions and little groups pressure was needed to convince a few individuals to reach full consensus in the IC groups.

For the case without evidence, the informal consensus (IC) groups demonstrated closer group agreement compared to the human computation (HC) groups. Opinions were more likely to shift when groups met face-to-face, as suggested by the study of Hutchings et al [18]. The choice of the degree of (dis)agreement as a process measure assumed that consensus is a good outcome and that for the case without evidence, IC groups fared better. Many guideline developers would disagree with the fact that consensus is a good outcome. We agree with that but we believe it was appropriate to use Kappa values as a process measure as reaching consensus is the primary goal of each consensus process.é

22-ii) Highlight unanswered new questions, suggest future research

"Future research is needed to confirm the results and to establish practical significance in a controlled setting of multidisciplinary guideline panels during real-life guideline development. "

Other information

23) CONSORT

The study was not registered because it did not concern a clinical trial, there were no patients involved. Although clinical guideline programmes could have an important impact on patients' health outcomes, this impact was not addressed in this pilot study to assess the feasibility of a new consensus method.

24) CONSORT

The full trial protocol can be obtained from the corresponding author

25) CONSORT

No funding was received.

X26-i) Comment on ethics committee approval

"Ethics committee approval for this study was obtained from the University Hospitals Leuven Medical Ethics Committee at December 2009. "

x26-ii) Outline informed consent procedures
"Informed consent to use the results for analysis was obtained offline from all participants."
X26-iii) Safety and security procedures
Not relevant in this proof of concept study of a new method for guideline development.
X27-i) State the relation of the study team towards the system being evaluated
"The authors and evaluators are the developers of the human computation application. There were no other conflicts of interest."
